# Hydrothermal approach to Co-Ni Layered Double hydroxide: high-performance electrode materials for energy storage devices

**DOI:** 10.1016/j.isci.2025.114031

**Published:** 2025-11-12

**Authors:** A. Manikandan, C. Ashwin, A. Dinesh, Senthilkumar Ramasamy, R. Mohan, Saravanan Rajendran, M. Santhamoorthy, S. Santhoshkumar, Prabhu Paramasvam, Sandeep Kumar, Gaurav Kumar

**Affiliations:** 1 Centre for Material Chemistry, Department of Chemistry, Karpagam Academy of Higher Education, Coimbatore, Tamil Nadu 641 021, India; 2 Department of Physics, Alagappa University, Karaikudi, Tamil Nadu 630 003, India; 3 Center of Excellence in Advanced Materials and Green Technologies, Amrita School of Engineering, Amrita Vishwa Vidyapeetham (Coimbatore Campus), Coimbatore, Tamil Nadu 641 112, India; 4 Department of Chemistry, K. Ramakrishnan College of Engineering (Autonomous), Affiliated to the Anna University, Samayapuram, Trichy, Tamil Nadu 621112, India; 5 PG & Research Department of Physics, Sree Sevugan Annamalai College, Devakottai 630 303, India; 6 Instituto de Alta Investigación, Universidad de Tarapacá, Arica 1000000, Chile; 7 School of Chemical Engineering, Yeungnam University, Gyeongsan 38541, Republic of Korea; 8 Department of Biochemistry, Saveetha Medical College and Hospital, Saveetha Institute of Medical and Technical Sciences, Chennai, Tamil Nadu, India; 9 Department of Mechanical Engineering, Mattu University, Mettu 318, Ethiopia; 10 Centre for Research Impact & Outcome, Chitkara University Institute of Engineering and Technology, Chitkara University, Rajpura, Punjab 140401, India; 11 Research and Innovation Cell, Rayat Bahra University, Mohali, Punjab 140301, India; 12 Research and Innovation Cell, Rayat Bahra University, Distt. Solan, Waknaghat, HP, India

**Keywords:** Applied sciences, Engineering, Energy storage

## Abstract

In this work, a binder-free Co-Ni layered double hydroxide (Co-Ni LDH) electrode with a distinct flower-like nanostructure was directly deposited on conductive nickel foam via a simple hydrothermal method. Structure, vibration, and electrochemical research were used to analyze the synthesized Co-Ni LDH. Moreover, the electrochemical effectiveness has been evaluated by electrochemical impedance spectroscopy (EIS), galvanostatic charge-discharge (GCD), and cyclic voltammetry (CV). The maximum capacitance of the Co-Ni LDH electrode reached 465 F/g with a current of 3 A/g. It also showed good cyclic stability, retaining 89% of its original capacitance following 8000 charge-discharge cycles. A Co-Ni LDH//AC electrode showed high specific capacitance (99.2 F/g at 2 A/g), power density (844 W/kg), and energy density (30.27 Wh/kg). Furthermore, the electrode retained 69.4% of its specific capacitance after a long cycle test (4000) in 3 M KOH solution. The combination of a binder-free design, direct growth, and flower-like nanoarchitecture represents a significant advancement in the development of cost-effective and scalable energy storage materials.

## Highlights

• Co-Ni LDH with a distinct flower-like structure was synthesized by the hydrothermal method
• The capacitance of the Co-Ni LDH electrode reached 465 F/g with a current of 3 A/g
• Cyclic stability 89% of its original capacitance following 8000 charge-discharge cycles
• Co-Ni LDH//AC electrode showed high specific capacitance 99.2 F/g at 2 A/g

## Graphical abstract

**Figure:**
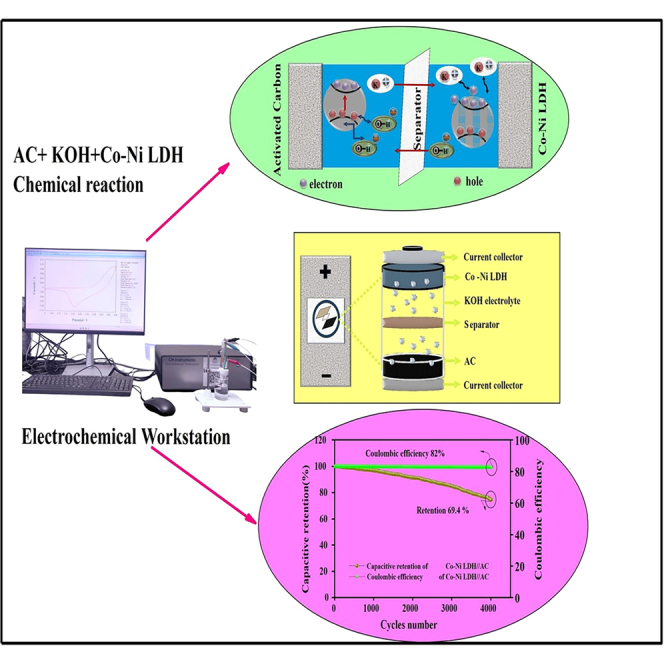

## Introduction

The beneficial features of supercapacitors (SCs), a sustainable source of energy device, such as their durability, substantial power density, and lightning-fast recharging or velocity, are responsible for their wide acceptance in electronics, smartphones, electrical systems, energy conservation, RAM backup devices, and power station arrays in recent years.^1^^,^^2^^,^^3^ Several types of SCs can be distinguished according to how they store charge. Electrical double-layer capacitors (EDLCs) have a low capacitance but a high-power density and stability, due to electrostatic charge dispersion and accumulation at the electrode/electrolyte interface.^4^^,^^5^^,^^6^ As a result of their distinct charge-storage processes, two types of SCs have been identified: electrical double-layer capacitors (EDLCs) and pseudocapacitors. The best substrates for EDLC electrodes are carbon-based materials, such as reduced graphene oxide (rGO), carbon quantum dots, carbon nanotubes, and activated carbon in particular, since they are easy to find, low-cost, and easy to obtain in a range of forms.^7^^,^^8^

The specific capacitance of EDLC with carbon electrodes is greater than that of other materials, and the effectiveness of EDLC comes from the charge accumulating on the electrode due to a physical electric field, which helps separate charges from the electrode-electrolyte interface.^9^ Materials based on metal oxides such as NiO, Bi_2_WO_4_, ZnO, Cr_2_O_3_, Mn_2_O_4_, NiCo_2_O_4_, V_2_O_3_, and Co_3_O_4_ are used as the elecrode for supercapacitors.^10^ The fact that charges are held in redox ions distinguishes porous carbon from pseudocapacitive processes, as demonstrated by metal oxide materials. Transition metal oxides including supercapacitor materials such as ruthenium oxide, manganese oxide, cobalt oxide, and nickel oxide, because the capacitance of metal oxide electrodes is primarily, due to pseudocapacitance, which is brought on by the movement of electrical charges during redox reactions.^11^^,^^12^^,^^13^

In this manner, hybrid supercapacitors (HSCs) are created for uses that require high energy and high-power capabilities at the same time.^14^ By mixing the electrodes of batteries and supercapacitors, the HSC shows the advantages of each technology, making it the ideal choice for applications including electric vehicles, high-power portable devices, and backup energy systems. Battery electroactive materials must have high activity, rate performance, and reliability during cycling since the best performance of HSC depends on the ideal match between the battery electrode and the supercapacitor electrode.^15^^,^^16^^,^^17^^,^^18^

Nickel hydroxide (Ni(OH)_2_) is one possible active electrode material for supercapacitors. During the charge process, Ni(OH)_2_ is converted into NiOOH (Ni(OH)_2_→NiOOH+H^+^+e^−^) to store energy and has unique properties (high specific energy, power density, and significant cyclability).^19^ The crystal structure, size, and shape of Ni(OH)_2_ govern its supercapacitive performance, which can be increased at the nanoscale.^12^^,^^20^

Cobalt species materials such as Co(OH)_2_, CoOOH, and Co_3_O_4_ have received a lot of interest in recent years, because of their superior electrochemical reversibility and cycle life, as well as their high capacitance.^21^^,^^22^^,^^23^^,^^24^

Along with LDHs, composite based metal oxides, have been deemed promising materials for the formation of a range of appealing nanostructured material electrode components, including porous carbon, hydroxide-type metal oxides, and metal sulfides. The goal of the current and future plan is to provide readers with a thorough understanding of the basic principles, innovative electrode materials, and modern wearable and flexible supercapacitor device designs,^25^^,^^26^^,^^27^^,^^28^^,^^29^ and to utilize a cost-effective and scalable method to fabricate Co-Ni LDH-based nanostructures with favorable electrochemical properties. In this present study, it was discussed a detailed justification for selecting this method was discussed, along with a comparison of its advantages, such as simplicity, low energy requirements, and suitability for large-scale production over more complex or less eco-friendly alternatives. Suggest future research avenues that could enhance the functionality and application range of LDHs and composite metal oxide-based materials, particularly in renewable energy solutions.

## Results and discussion

### XRD analysis

**Figure 1:**
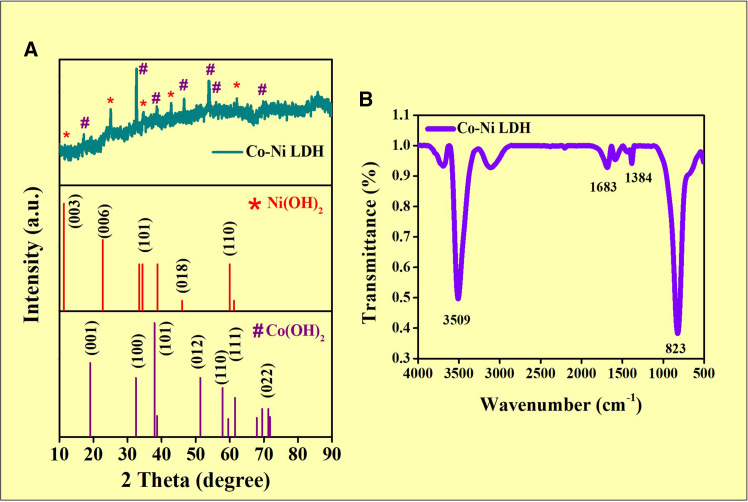
XRD and FTIR spectral analysis (A) XRD pattern of Co-Ni LDH (B) FTIR spectrum of Co-Ni LDH.

The crystal structure of Co-Ni LDH had been identified by XRD pattern analysis. The XRD pattern of Co-Ni LDH’s are shown in Figure 1A. The Ni(OH)_2_ crystals were discovered at 2*θ* angles of 11.6°, 23.9°, 34.9°, 45.6°, and 60.5°, and the corresponding crystal planes are (003), (006), (101), (018), and (110), respectively, all represented by these angles (JCPDS Card 00-038-0715). Likewise, Co(OH)_2_ crystals were found at 2*θ* angles of 19.0°, 32.4°, 51.9°, 58.2°, 61.4°, and 71.3°, and the corresponding crystal planes are (001), (100), (012), (110), (111), and (021), respectively, matched well with the JCPDS Card 00-030-0443.^30^ Depending on the areas of high intensity peak, the dimension of the crystallite was identified by the Debye-Scherrer equation.^31^^,^^32^

(Equation 1) $$D=\frac{0.9\times \lambda }{\beta \times \cos\;\theta }$$

The λ is the wavelength of the light (1.5406 Å), the full-width half maximum (*β*), and the degree of diffraction angle (*θ*). We discovered that 18 nm was the average crystal size.

### FT-IR studies

Figure 1B shows the FT-IR spectra of Co-Ni LDH nanocomposites. O-H stretching band peak at 3509 cm^−1^, which is brought about by metal-hydroxyl compounds.^33^ One is able to connect the band with its center at 1683 cm^−1^ to the twisting vibration of water. It is also likely for one to attribute the shaking of layers CO_3_^2−^ and NO_3_^−^, the anions, to the spectrum at 1384 cm^−1^.^34^ With concerted debt instruments, CO_3_^2−^ united Ni^2+^ and Co^2+^ ions and created the nickel-cobalt carbon alkaline hydrate; however, NO_3_^−^ lingered in the LDH interlayer. One can link the large height at 600-850 cm^−1^ to the interaction (M = Co and Ni) vibrational.^34^^,^^35^

### FESEM analysis

FESEM analysis (Gemini 300 Zeiss) was carried out to investigate the morphological features of Co-Ni LDH. Figure S1A shows the empty nickel foam image, and similarly, the FESEM images at different magnifications in Figures S1B**–**S1F. Figure S1B presents FESEM images of the electrodeposited nanostructures as observed from the top. FESEM analysis reveals that the Co-Ni LDH is uniformly deposited on the 3D porous structure of the nickel foam, forming a well-defined flower-like nanostructure shown in Figure S1C. The images reveal a uniform growth of densely packed nanosheets on the nickel foam skeleton, creating a three-dimensional hierarchical structure Figures S1D–S1F. This hierarchical morphology provides a large electroactive surface area and interconnected pathways, which facilitate efficient ion/electron transport, key factors enhancing the electrochemical performance for supercapacitor applications (Figure 5). The EDAX spectra and the elemental composition data are shown in Figures S1G–S1L. EDX mapping of the composite materials revealed a high degree of purity and the absence of contaminants. EDX mapping images Figures S1G–S1J show the elemental distribution. A uniform particle distribution across the whole Co-Ni LDH region is demonstrated by the arrangement of components Co, Ni, and O. The weight proportion of the as-prepared Co-Ni LDH was 8.36% of Co, 56.6% of Ni, 4.6% of C, and 30.4% of O, according to elemental mapping, as seen in Figures S1K and S1L.

#### Synergistic effects

The combination of these factors creates a synergistic effect that greatly enhances the electrochemical performance of Co-Ni-LDH. Improved conductivity, enhanced redox activity, greater structural stability, increased surface area, and better ion transport all contribute to a material that is highly efficient for charge storage. This makes Co-Ni-LDH an excellent candidate for use in supercapacitors, which require materials with high power density, long cycle life, and stable performance under various operating conditions. These improvements make Co-Ni-LDH a promising material for advanced energy storage applications, offering enhanced conductivity, stability, redox activity, and ion transport capabilities.

#### Interlayer spacing

The layered structure of Co-Ni LDH provides a high surface area and facilitates electrolyte ion diffusion between layers. Increased interlayer spacing (as indicated by XRD) enhances ion accessibility and contributes to higher pseudocapacitive behavior.

#### Crystal orientation

SEM and XRD analyses suggest preferential growth along specific crystallographic planes, which can promote rapid electron transport and improve redox kinetics during cycling.

#### Defect density

The presence of defects, as suggested by broad XRD peaks, can introduce additional active sites for redox reactions, enhancing the charge storage capability.

### HR-TEM and SAED analysis

**Figure 2:**
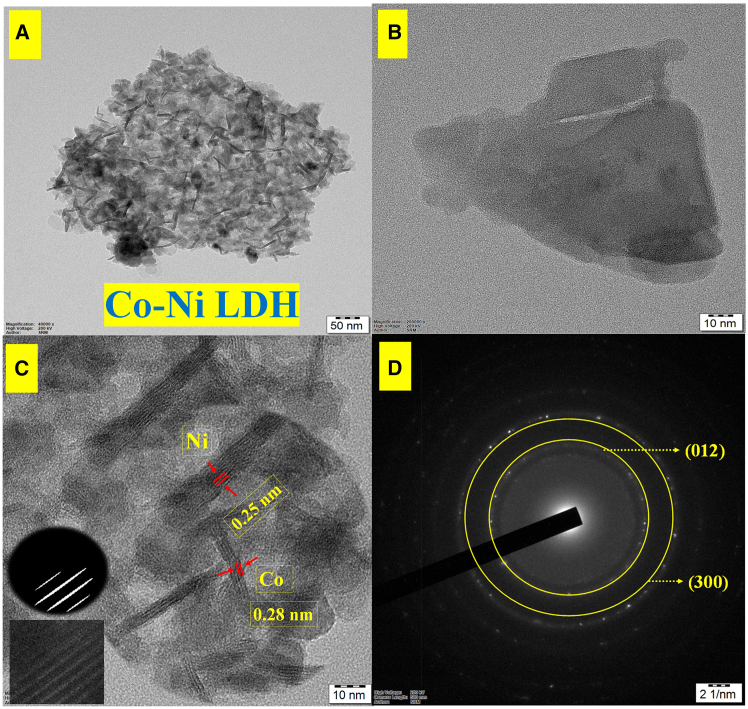
TEM and SAED patterns of Co-Ni LDH (A–C) HR-TEM images of Co-Ni LDH and (D) SAED pattern of Co-Ni LDH.

The transmission electron microscope, TEM (JEM-2100 plus) and HR-TEM images are shown in Figures 2A–2C, featuring superior resolution, is being used to evaluate the arrangement of lattices and morphological particle size, surface nature, and d-spacing of Co-Ni LDH. The lattice spacing values of 0.25 nm for Ni(OH), and 0.28 nm for Co(OH) is shown in Figure 2C. Additionally, the lattice planes observed from the selected area electron diffraction (SAED) rings were (012) and (300), respectively, as shown in Figure 2D. The Co-Ni LDH crystalline is further confirmed by XRD pattern and these results agree well.^22^^,^^36^

### Three-electrode setup electrochemical analysis

**Figure 3:**
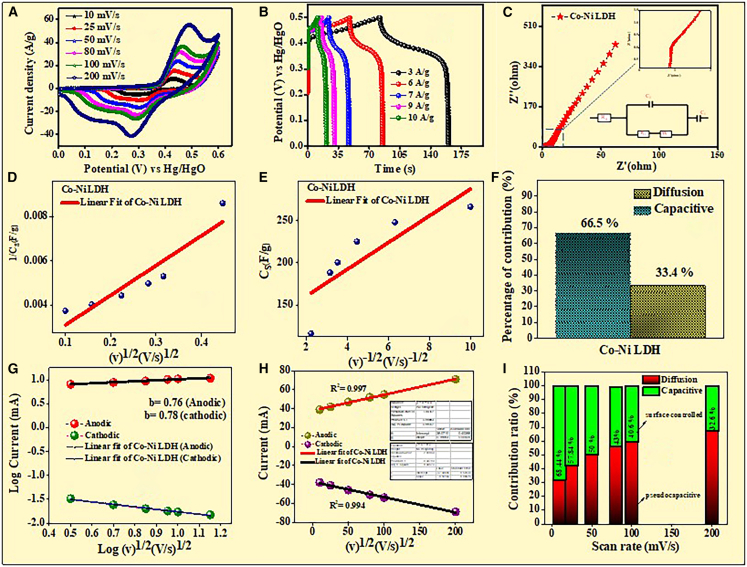
CV analysis in a three-electrode system (A) CV spectra, (B) GCD analysis, (C) EIS spectra, (D) Total capacitance (v^1/2^ vs. C_s_^−1^), (E) outer capacitance (v^−1/2^ vs. C_s_), (F) capacitive-diffusion plot, (G) determination of b value from the plot log(current) versus log(scan rates), (H) a linear relationship is found between the redox peak current and the square root of the scan rate, and (I) Dunn plot of Co-Ni LDH.

Cyclic voltammetry (CV) analysis (CHI660 E) was used for examining the electrochemical behavior of Co-Ni LDH, and Figure 3A displays the outcomes. Either the material’s faradaic or non-faradaic behavior has been evaluated using a CV graph. A standard three-electrode setup in the research studies was carried out using a solution containing 3 M potassium hydroxide electrolyte.^37^ This design makes use of Hg/HgO as a reference electrode, a Co-Ni LDH active electrode, and a platinum counter electrode. Using scan speeds of 10, 25, 50, 80, 100, and 200 mV/s, this cell was operated throughout the 0–0.6 V voltage limit. Using the following formula, the three-electrode system’s trio of electrode specific capacitance (Cs) was determined^38^:

(Equation 2) $$C_{1}=\frac{\int I\;\left(V\right)dV}{m_{1}\nu *\Delta v}$$

(Equation 3) $$C_{2}=\frac{I\times \Delta \;t}{m\;\times \;\Delta v}$$

Where m_1_ signifies the mass (g) of the active material coated on Ni foam, I(V) means the current density at the specific capacitance (F/g) discovered from CV and GCD experiments, and C_1_ and C_2_ indicate the discharge duration (s) given a sweep voltage (V). The applied scan rate is denoted by v (mV/s), and the CV’s potential is indicated by Δv.

According to the CV analysis, the Co-Ni LDH specific capacitance (Cs_1_) values at scan rates of 10, 25, 50, 80, 100, and 200 mV/s were estimated to be 266.7, 248.1, 225.4, 200.5, 188.3, and 116 F/g, respectively (Figure 3A).

As further demonstrated by the GCD curve of the Co-Ni LDH generated via a simple hydrothermal procedure in Figure 3B, current densities ranging between 3, 6, 7, 9, and 10 A/g in the potential window of 0–0.5 V are thoroughly investigated. With a 3 M KOH electrolytic solution, the three-electrode design was investigated for the charging mechanism studies.^39^ Additionally, investigations on galvanostatic charging and discharge were used. According to Figure 3B, the discharge duration of Co-Ni LDH is longer (465, 447, 315.9, 268.2, and 194 F/g at 3, 6, 7, 9, and 10 A/g, respectively).

The impedance spectra reveal Co-Ni LDH as well as the resistance of the solution (R_s_) and the transmission of charge (R_ct_). According to Figure 3C, the Nyquist plot produces R_s_ and R_ct_ values of 0.6 and 0.8 Ω, respectively, demonstrating their exceptional charge transfer efficiency. Similarly, the equivalent circuit shows low R_s_, R_ct_, and Warburg impedance (W), indicating the improved capacitance behavior of Co-Ni LDH.^40^ The relative amounts of electric double-layer capacitance (EDLC) and pseudocapacitive (PC) behavior have been assessed using Trasatti and Dunn’s methods to measure the charge-collecting process of an electrode. By extending the Cs to v = 0, the proportionality of some kind of capacitance (1/Cs) can be displayed in relation to the scan rates’ square root (v^1^/^2^) to determine the electrode’s total capacitance.^41^

(Equation 4) $$1/C_{s}\left(V\right)=\text{const}\;v^{1/2}+1/C_{\text{total}}$$

The total charge (C_total_) that has been collected across the electrode can be estimated through the combination of the charges maintained at the outermost portion of the electrode (C_outer_) and its inner portion (C_inner_).

(Equation 5) $$C_{\text{outer}}+C_{\text{inner}}=C_{\text{total}}$$

The C^−1^ vs. k^1/2^ plot at k = 0 and the C vs. k^−1/2^ graph at k = ∞ were used to determine the sum of charge generated at the electrode’s surface and charge stored beyond its boundaries. Using the method previously described, the total capacitance (C_total_) of Co-Ni LDH has been estimated to be 384.61 F/g, as seen in Figure 3D.

In addition, Figure 3E shows that the outer capacitance (C_outer_) of Co-Ni LDH was found to be 128.73 F/g 255.8 F/g was the charge stored at the (C_inner_) in respect to Co-Ni LDH. The charge stored at the electrode can be explained by the combined contribution of diffusion-controlled and capacitive-controlled processes. The expression for capacitively controlled reactions is supplied by^42^:

(Equation 6) $$Q_{i}\;\left(\%\right)=\frac{q_{i}}{q_{T}}\times 100\;\%$$

(Equation 7) $$Q_{o}\;\left(\%\right)=\frac{q_{o}}{q_{T}}\times 100\;\%$$

As shown in Figure 3F, the percentage of diffusion-controlled charge (C_outer_ %) for Co-Ni LDH was found to be 33.47%. It was discovered that 66.5% of the electrode’s charge was capacitive-controlled (C_inner_ %). The charge storage behavior was affected by the parameter ‘b'; a value of b ≤ 1 indicated electrode materials of the capacitive type, whilst a value of (anodic) peak 0.76 and cathodic peak 0.78 indicated pseudocapacitive properties (see Figure S2). The link between log(i) and log(v) is seen in Figure 3G, where the graph’s slope produces a capacitive behavior. Figure 3H shows the R^2^ value nearer to 1 for both anodic and cathodic peaks, indicating that reversible faradaic reactions are stable, thus confirming that the electrode material is battery-type and capacitor-type. To support this, the power law was utilized, which indicated the relationship between the sweep rate and peak current, expressed as^43^

(Equation 8) $$i={\text{av}}^{b}$$

**Figure 4:**
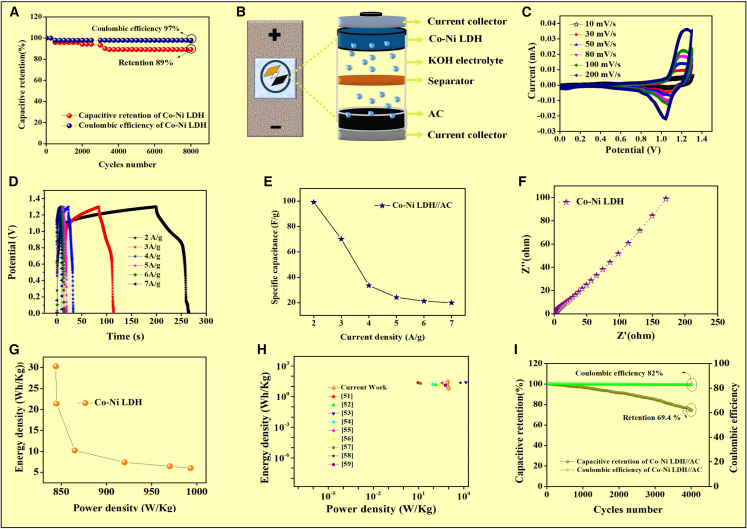
CV analysis in a two-electrode system (A) capacitive retention and coulombic efficiency of Co-Ni LDH, (B) Schematic diagram of Co-Ni LDH HSC, (C) CV analysis, (D) GCD analysis, (E) specific capacitance versus current rate, (F) EIS spectra, (G) Ragone plot, (H) comparison of energy vs. power density, and (I) capacitive retention and coulombic efficiency of Co-Ni LDH//AC hybrid supercapacitor device.

Equation 8 relative contributions of pseudocapacitance and electric double-layer capacitance (EDLC) are crucial for a comprehensive evaluation of the charge storage mechanism. In response, we conducted cyclic voltammetry (CV) measurements at various scan rates (10 mV/s - 200 mV/s). By applying the power law relationship (i = aν^b^) and further deconvoluting the current response using Dunn’s method, we have quantified the capacitive (surface-controlled) and diffusion-controlled (pseudocapacitive) contributions. These results have now been included in the revised article in Figure 3I, along with a detailed discussion and the corresponding Figure 3F. The IR drop that was observed in the three-electrode material to achieve these two strategies was employed (i) to have perfect mass balance between the dissimilar electrodes, and (ii) the separator used in the Whatman filter paper was soaked in a 3 M KOH electrolyte with high wettability. But the main reason for the IR drops in our material, such as GCD analysis, strongly suggests that the ion moves continuously in electrolytes in different current densities sometimes (KOH). Drying after the current density response results in poor performance. The Co-Ni LDH electrodes exhibited good capacitive retention of 89%, cyclic stability, and a high coulombic efficiency of 97% at a current density of 10 A/g, even after 8000 cycles Figure 4A.

### Electrochemical analysis of the hybrid device

The whole-cell configuration and the potential range of 0.0–1.3 V in 3 M KOH electrolyte were used to assess the performance of an asymmetric supercapacitor (ASC) device with a manufactured Co-Ni LDH NPs positive electrode and an activated carbon (AC)-based negative electrode Figure 4B.

Co-Ni LDH//AC electrode-associated CVs are analyzed at a constant scan rate of 10 and 200 mV/s, as shown in Figure 4C.^24^ The significant detrimental faradaic contributions, such as deterioration, substance loss of weight, and electrolyte dissociation/or deterioration, would also affect the device’s longevity. If you want to apply the equation, another requirement is to keep the energy (Q) equal on both cathode and anode electrodes.^24^

(Equation 9) Q=V×Cp×ma

In which V resides the constant, the potential window, while Cp is the electrode’s specific capacitance, where ma is the mass of the material used for the active electrode that operates across the V range. As a result, the positive electrode’s mass ratio to the negative electrode was kept at the theoretical mass ratio calculated, given in the equation.^39^^,^^44^

(Equation 10) $$m{◦}^{+}/m{◦}^{-}=\left(C^{-}\times \Delta V^{-}\right)/\left(C^{+}\times \Delta V^{+}\right)$$

According to Equation 10, a perfect balance of mass is a ratio of 1:1. C+ and C− stand for the exact capacitance of the positive and negative electrodes, m◦+ and m◦− for their masses, and ΔV+ and ΔV− for the associated changing potential. The chemical in question has a 3-milligram total deposition in a two-electrode setup. The mass ratio of the Co-Ni LDH (positive electrode) to activated carbon (AC, negative electrode) was carefully optimized based on the charge balance principle (m^+^ = m^−^), where Q = C × ΔV × m (C: specific capacitance, ΔV: potential window, m: mass of active material). The specific capacitance values and potential windows of both electrodes were experimentally determined in a two-electrode setup. Based on these data, we calculated and adjusted the mass loading of each electrode to ensure charge balance, which is critical for maximizing energy storage performance and device stability.

In any commercial supercapacitor, a mass loading of 3–8 mg is highly recommended. Hence, the same material was tested for a higher mass loading of 3 mg in a three-electrode setup using Ni-foam as the substrate. A specific capacitance of 465 F/g was achieved at 3 A/g. The experimental setup details are provided in the Figure 3B. However, the practical difficulties in using a liquid electrolyte commercially fabricated is using a KOH electrolyte. The mass loading on both the nickel foams (1 × 1 each) was 1 mg and 0.8 mg, with a total loading of 1.8 mg. 3 M KOH was used as an electrolyte between the two electrodes of the device, and the synthesis procedure is provided in the supporting information. The device was sealed completely as shown in Figure S2. Figure 4C displays the CV curves of the built Co-Ni LDH//AC asymmetric supercapacitor, which show values of 88.45, 49.8, 39.23, 31.2, 26.9, and 22.4 F/g at scan speeds of 10, 30, 50, 80, 100, and 200 mV/s across the potential within 0–1.3 V.^31^

The Co-Ni LDH//AC HSC’s charge-discharge characteristics were examined by taking GCD profiles at varying concentrations of current ranging from 2 to 7 A/g within a potential window of 0–1.3 V. In Figure 4D, the typical nonlinear charge-discharge curves show the charge stored by the slow faradaic redox process.^36^ According to the GCD curves at different current densities, the Co-Ni LDH//AC HSC device has specific capacitances are 99.23, 70.15, 33.53, 24.2, 21.23, and 19.9 F/g at 2, 3, 4, 5, 6, and 7 A/g, as shown in Figure 4E. We agree that the noticeable drop in specific capacitance from ∼99 F/g in the device is significant and warrants further discussion.

#### Electrode mismatch

In the asymmetric device Co-Ni LDH//AC, the positive and negative electrodes are composed of different materials with distinct electrochemical behaviors. Any mismatch in their capacitive performance can lead to inefficient charge storage and reduced overall capacitance.

#### Mass imbalance

While efforts were made to balance the mass of the electrodes based on their specific capacitances, slight deviations during electrode fabrication can cause an imbalance, affecting the charge storage capability of the device.

#### Diffusion limitations

In a two-electrode configuration, ion diffusion pathways are more restricted due to the presence of a separator and tighter packaging compared to the three-electrode system, which may lead to decreased ionic mobility and reduced utilization of active materials.

#### Internal resistance

The full device includes additional components (current collectors, separators, and so forth) that may increase internal resistance, contributing to lower effective capacitance.

A small semicircle and a straight line were visible in the EIS spectra. A semicircle shows the Co-Ni LDH//AC’s charge transfer resistance phenomena, whereas a straight line shows the electrodes’ redox characteristics. Figure 4F shows that the exceptionally low estimated values of rapid ion transfer were assisted by the (Rs) resistance of the solution and the (Rct) charge-transfer resistance. The Rs and Rct values are 0.8 Ω and 1.8 Ω, respectively.

The device delivers a high energy density of 30.27 Wh/kg and a power density of 844 W/kg, calculated based on the total mass of the active materials at both electrodes. The power density (P_cell_) and energy density (E_cell_) of the Co-Ni LDH//AC HSC device were assessed by.^45^^,^^46^

(Equation 11) $$P_{\text{cell}}=3600\times \frac{E_{cell}}{\Delta t}$$

(Equation 12) $$E_{\text{cell}}=\frac{1}{7.2}C_{2\text{HSC}}{\left(\Delta V\right)}^{2}$$

where.

• Ccell is the specific capacitance of the full cell in F/g,
• ΔV is the voltage window in volts,
• Δt is the discharge time in seconds,
• The factor 3.6 is used to convert Wh to J.

These values are normalized to the total mass of active materials from both the positive and negative electrodes.

The Ragone plot of the Co-Ni LDH//AC ASC device, measured within the potential window of 0–1.3 V, is displayed in Figures 4G and 4H. As expected, its energy and power density (2 A/g) are extremely high, with 30.27 Wh/kg and 844 W/kg, respectively. At their respective current densities of 3–7 A/g, the Co-Ni LDH||AC hybrid device had higher E_cell_ values (21.4, 10.23, 7.39, 6.47, and 6.0 Wh/kg) and higher P_cell_ values (865, 920, 933, 970, and 993 W/kg). It was found that these results exceeded those of the previous study (Table 1).^47^^,^^48^^,^^49^^,^^50^^,^^51^^,^^52^^,^^53^^,^^54^^,^^55^^,^^56^^,^^57^^,^^58^^,^^59^ The device exhibits remarkable cyclic stability, retaining 69.4% of its coulombic efficiency and 82% of its capacity even after 4000 cycles in Figure 4I.

**Table 1:** Comparison of the Ragone plot of the Co-Ni LDH//AC HSC Device with Previously Reported Literature

Materials	Electrolyte	Energy density (Wh/kg)	Power density	Reference
Sol-gel synthesis of CuFe_2_O_4_	6 M KOH	10.23	898.2 W/kg	Nikam *et al*.^47^
Co_3_O_4_/CMC nanoflakes	2 M KOH	18	1543 W/kg	Babu *et al*.^48^
CoFe_2_O_4_//AC ASC	3 M KOH	22.85	6000 W/kg	Nwodo *et al*.^49^
Nanoporous NiO Films as SCs	3 M KOH	16.5	89 W/kg	Liang *et al*.^50^
3D hierarchical pompon-like cop hollow microspheres	6 M KOH	22.2	374.9 W/kg	Ding *et al*.^51^
Ag-decorated MnO_2_ HMSS//AC	1 M Na_2_SO_4_	15.9	250.3 W/kg	Yi *et al*.^52^
CNF/graphene and Carbon Aerogel with MnO_2_	1 M Na_2_SO_4_	20.0	13.5 W/kg	Shalabh^53^
Honeycomb b-like mesoporous NiO	6 M KOH	23.5	495 kW/kg	Ren *et al*.^54^
Spinel ferrite sol-gel auto combustion method	3 M KOH	30	301 W/kg	Sutka and Mezinskis^55^
MnO_2_-Carbon black//AC Suspension ASC	2 M KOH	11	50 W/kg	Li *et al*.^56^
AC//MnO_2_/CNTs	1 M Na_2_SO_4_	13.3	600 W/kg	Subagio *et al*.^57^
Alpha-Ni(OH)_2_ Nanosphere as high-performance SCs	2 M KOH	26.5	0.82 kW/kg	Aghazadeh *et al*.^58^
Self-doped Co_3_O_4_	6 M KOH	29.5	104 W/kg	Li *et al*.^59^
Co-Ni LDH	3 M KOH	30.27	844 W/kg	Present work

We acknowledge that demonstrating mechanical flexibility is essential to validate the potential of the device for flexible and wearable electronics. In response, we have now conducted bending tests at room temperature while monitoring electrochemical performance. The device retained stable capacitance with negligible degradation under repeated bending, confirming its mechanical robustness. These results and the corresponding discussion have been added along with photographic and video evidence and performance data attached to Figures S3 and S4.

### Conclusion

A straightforward hydrothermal process was used to successfully prepare the scalable Co-Ni LDH. Investigations using XRD, FT-IR, HR-TEM, and SAED patterns verified the structural and vibrational features. Using CV, GCD, and EIS analysis, the electrochemical properties in aqueous electrolyte solutions having 3 M potassium hydroxide were studied. Co-Ni LDH demonstrated a notable capacitance of 465 F/g at a scan flow rate of 3 A/g. A good capacitance retention of 89% is revealed by CV measurement after 8000 cycles. Due to an EIS spectrum, the charge transfer was 0.8 Ω, and the solution resistance was 0.6 Ω. It has ultra-high energy and power density (2 A/g) of 30.27 Wh/kg and 844 W/kg, respectively, and 69.4% high-capacity retention over 4000 cycles was demonstrated by the hybrid Co-Ni LDH//AC device.

### Limitations of the study

The Co-Ni layered double hydroxide made through hydrothermal synthesis and the materials showed very good electrochemical activity, but it definitely has some limitations that need to be considered. The electrochemical measurements were performed in 3 M KOH solution using a three-electrode system and further validated in the asymmetric device itself. These standard conditions are widely used for testing, but they may not show the actual performance of full-cell devices under different temperatures, pH levels, or non-aqueous electrolytes needed in industry. Further, the testing conditions themselves may not reflect the real working environment of these devices. Basically, the device testing showed that capacitance drops at higher currents, and cycling stability is moderate with around 69% retention after 4000 cycles. The same pattern indicates decent but not excellent long-term performance. This shows possible problems regarding ion-diffusion limits, electrode/electrolyte interface damage, and partial structural breakdown of the LDH as per prolonged operation conditions. Basically, the energy and power densities were calculated using only the active mass, which means the same calculations did not include current collectors, binders, separators, or packaging materials. As per the structural analysis, post-cycling tests were not done, leaving doubts regarding the chemical or shape changes that caused performance loss. As per these studies, critical insights can be obtained regarding redox mechanisms and phase stability. Moreover, the hydrothermal method works well in laboratories, but surely faces problems such as inconsistent results, uneven batches, and high costs when used for large-scale electrode production. The study actually did not address environmental and safety issues such as the long-term effects of using cobalt and nickel. It definitely missed discussing how strong alkaline solutions can cause corrosion problems. As per the requirements, these aspects are essential regarding sustainable and safe use in next-generation energy storage systems. Future studies should actually focus on improving devices, monitoring structures during use, engineering better electrolytes, and developing clean synthesis methods. These investigations will definitely be necessary to convert promising lab results into practical real-world applications.

## Resource availability

### Lead contact

Requests for further information and resources should be directed to and will be fulfilled upon reasonable request by the lead contact, Professor Dr. A. Manikandan (manikandan.frsc@gmail.com).

### Materials availability

This study did not generate new, unique reagents.

### Data and code availability

• Non-confidential data reported in this study can be requested by contacting the lead author upon reasonable request.
• This article does not report the original code.
• Any non-confidential additional information required to reanalyze the data reported in this article is available from the lead contact upon reasonable request.

## Acknowledgments

The authors are thankful to the Centre for Material Chemistry, Karpagam Academy of Higher Education, Coimbatore – 641021, Tamil Nadu, India, for providing the necessary facilities to synthesize the samples and to carry out electrochemical studies. The authors also acknowledge Amrita Vishwa Vidyapeetham (Coimbatore Campus), Coimbatore – 641112, and the Nanotechnology Research Centre (NRC), SRM Institute of Science and Technology (SRMIST), Tamil Nadu, India, for providing FT-IR, XRD, SEM, and TEM analysis facilities. Furthermore, the authors are grateful to the DST-SERB research project scheme [File No. EEQ/2023/001035], New Delhi, India, for financial support.

## Author contributions

A.M. and C.A. conceived the study, developed the methodology, performed formal analysis and investigations, conducted the calculations and simulations, curated the data, wrote the original draft, and created visualizations. A.D. and S.R. coordinated and supervised the research direction. R.M., M.S., and P.P. contributed to the validation and article review. S.K. and G.K. provided supervision, secured project resources and funding, and participated in critical revisions of the article. All authors discussed the results and contributed to the final version of the article.

## Declaration of interests

The authors declare no competing interests.

## STAR★Methods

### Key resources table

**Table:** 

REAGENT or RESOURCE	SOURCE	IDENTIFIER
Nickel nitrate hexahydrate	SRL	13478-00-7
Urea	SRL	57-13-6
Cobalt nitrate hexahydrate	Merck, India	10026-22-9

### Method details

This section shows methodologies employed for synthesizing Co-Ni Layered Double Hydroxide, their electrode fabrication, physical characterization, and subsequent electrochemical evaluations.

#### Synthesis of Co-Ni LDH electrode on nickel foam

**Figure 5:**
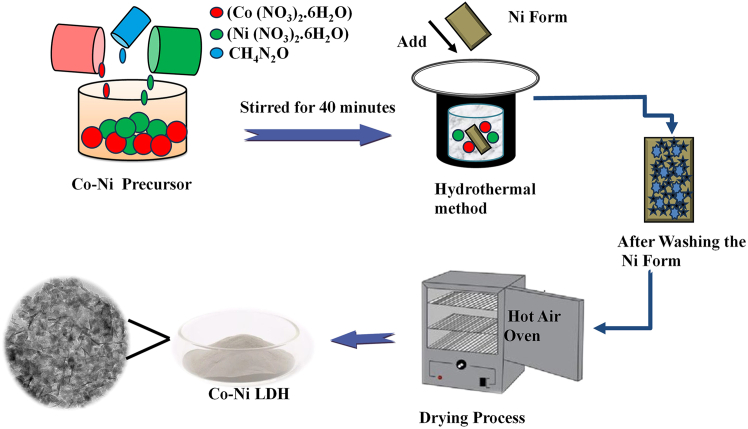
Synthesis process of Co-Ni LDH.

In a simple hydrothermal method, a post-drying treatment was used to create the Co-Ni LDH on Ni foam. The specific synthesis stages are as follows: First, nickel foam (1 × 1 cm^2^) was cleaned using a portion of absolute ethanol and deionized (DI) water, which was sonicated for 20 min. These were then mixed with 70 mL of DI water, 1 mmol cobalt nitrate hexahydrate, 6 mmol urea, and 2 mmol nickel (II) nitrate hexahydrate. The mixture was stirred for 40 min. Following this, the reaction mixture was moved to the Teflon-lined autoclave and filled with bits of recently cleaned nickel foam, then heated for 10 h at 120°C. Following that, the electrode is cleaned multiple times using purified water and ethanol. In the case of Figure 5, the conducting electrode was subsequently dried for one day, for consecutive hours at 80°C in a hot air oven.
