# Supplementary material for: Hydrothermal approach to Co-Ni Layered Double hydroxide: high-performance electrode materials for energy storage devices
Source: iScience. 2025 Nov 12;29(2):114031. doi: 10.1016/j.isci.2025.114031 (PMC12915251; doi:10.1016/j.isci.2025.114031)
Supplement: Document S1. Figures S1–S4 [file mmc1.pdf]

iScience, Volume *29*

Supplemental information

Hydrothermal approach to Co-Ni Layered Double hydroxide: high-performance electrode materials for energy storage devices

A. Manikandan, C. Ashwin, A. Dinesh, Senthilkumar Ramasamy, R. Mohan, Saravanan Rajendran, M. Santhamoorthy, S. Santhoshkumar, Prabhu Paramasvam, Sandeep Kumar, and Gaurav Kumar

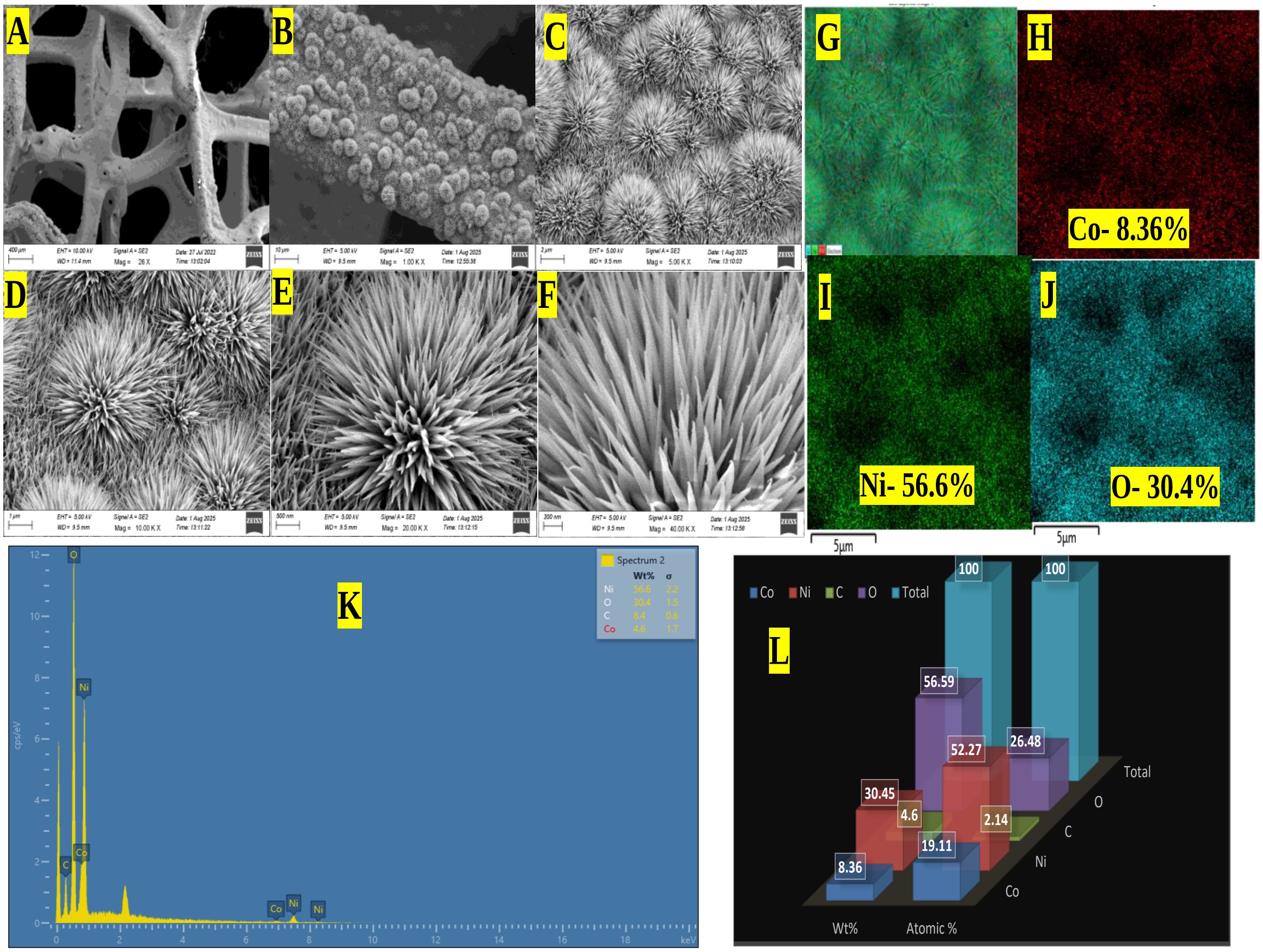

S1. SEM, elemental mapping and EDX spectral analysis of Co-Ni LDH.

(A) Ni foam, (B-F) FESEM images of Co-Ni LDH, (G-J) EDX mapping of Co, Ni, O, (K) EDX spectra, and (L) Bar diagram of Co-Ni LDH.

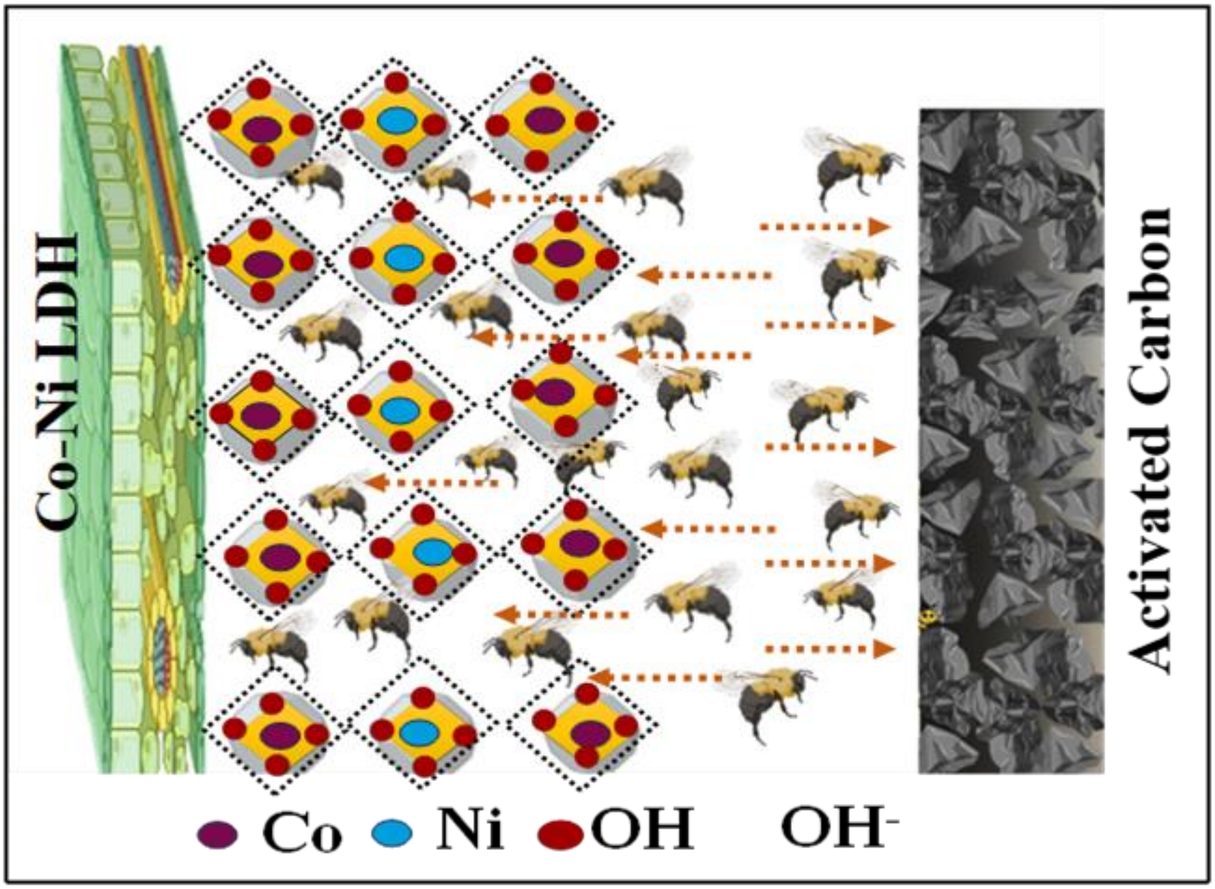

S2. Mechanism of charge storage in Co-Ni LDH.

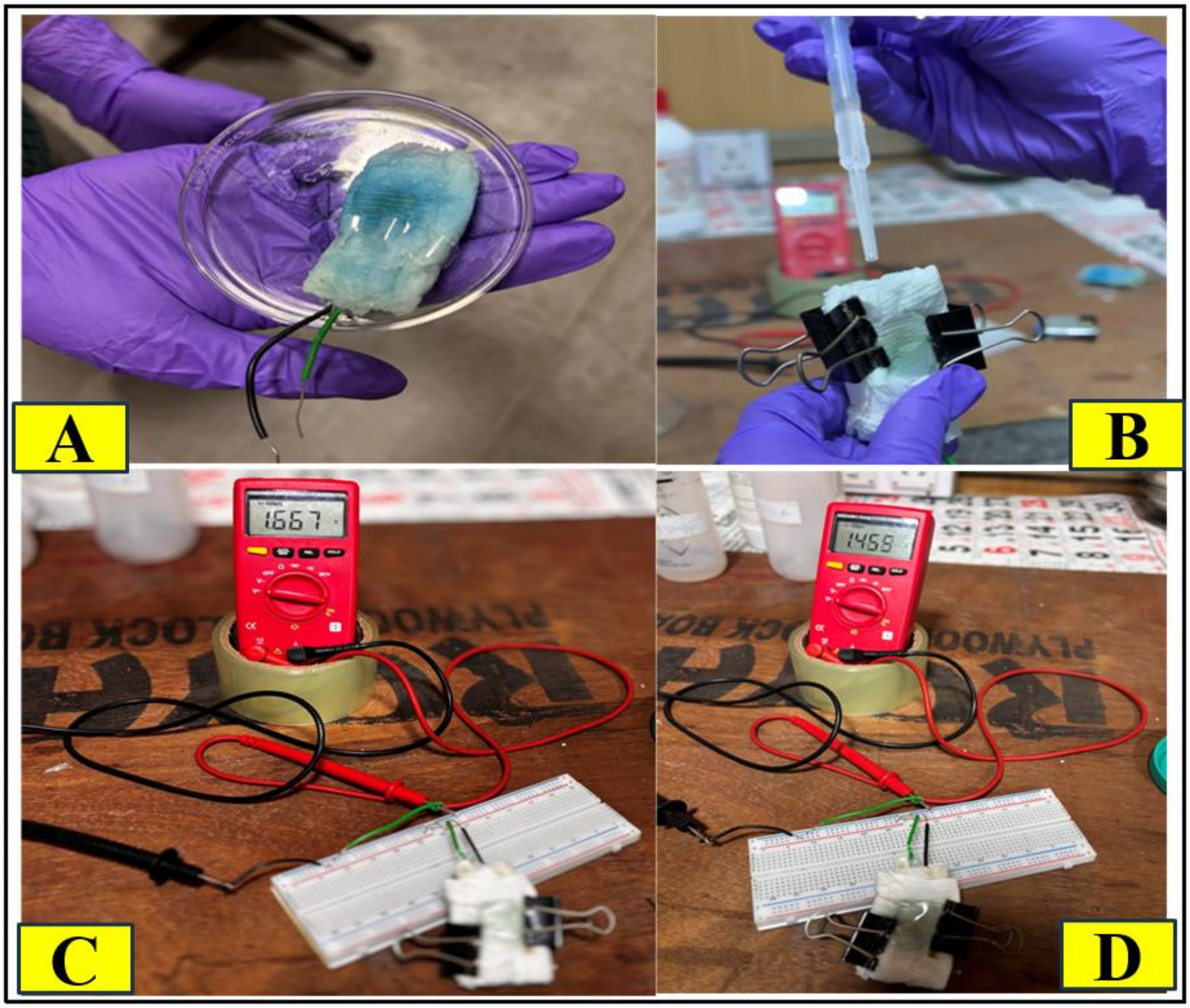

S3. CV analysis in a two-electrode system. CV analysis in a two-electrode system. (A) Model (two months before prepared device Co-Ni LDH//AC), (B) After KOH adding, and (C, D) discharging rate used in multimeter reading.

discharge.MOV

S4. The device is discharging a video clip in a multimeter.
